# College Changes

**Published:** 1903-05

**Authors:** 


### COLLEGE CHANGES.

With the closing of Harvard Veterinary Department and the prospective closing of the doors at Montreal, Canada, comes the announcement that the Veterinary Department of the Iowa Agricultural College at Ames, Iowa, will increase its curriculum to four years of eight months each. This extension of the course in the Veterinary Department we understand is in keeping with the courses in all the other departments of the State College. In conjunction with the change Dr. John H. McNeal has been selected as the Dean of the Veterinary Department. As we go to press we have not been able to learn what changes in the curriculum are to be made, or what additions to the teaching staff added to cover the extended course. We trust that a more liberal support by the State is to be accorded, more adequate buildings, and additions to the faculty to insure a proper measure of success for this the first American veterinary institution to adopt a four-year course.

From the new Administration in the city of Philadelphia the veterinary profession are looking forward to some much-needed reforms in meat and milk-inspection. The Keystone Veterinary Medical Association is active in urging this advancement.

